# Author Correction: Catechin and curcumin interact with S protein of SARS-CoV2 and ACE2 of human cell membrane: insights from computational studies

**DOI:** 10.1038/s41598-021-88218-3

**Published:** 2021-04-13

**Authors:** Atala B. Jena, Namrata Kanungo, Vinayak Nayak, G. B. N. Chainy, Jagneshwar Dandapat

**Affiliations:** 1 Centre of Excellence in Integrated Omics and Computational Biology, Utkal University, Bhubaneswar, Odisha 751004 India; 2 Post Graduate Department of Biotechnology, Utkal University, Bhubaneswar, Odisha 751004 India

Correction to: *Scientific Reports* 10.1038/s41598-021-81462-7, published online 21 January 2021

This Article contains a typographical error in the Introduction section where,

“SARC-CoV2, an enveloped single stranded positive sense RNA virus belonging to family *Coronaviridae* is a matter of global concern.”

Should read:

“SARS-CoV2, an enveloped single stranded positive sense RNA virus belonging to family *Coronaviridae* is a matter of global concern.”

